# Rasmussen Encephalitis: Clinical Features, Pathophysiology, and Management Strategies—A Comprehensive Literature Review

**DOI:** 10.3390/medicina60111858

**Published:** 2024-11-12

**Authors:** Ana Leticia Fornari Caprara, Jamir Pitton Rissardo, Eric P. Nagele

**Affiliations:** Neurology Department, Cooper University Hospital, Camden, NJ 08103, USA; fornari-caprara-ana@cooperhealth.edu (A.L.F.C.); nagele-eric@cooperhealth.edu (E.P.N.)

**Keywords:** rasmussen encephalitis, rasmussen syndrome, encephalitis, epilepsy, hypometabolism, epilepsia partialis continua, refractory epilepsy, cerebral hemiatrophy

## Abstract

Rasmussen encephalitis (RE) is a rare and progressive form of chronic encephalitis that typically affects one hemisphere of the brain and primarily occurs in pediatric individuals. The current study aims to narratively review the literature about RE, including historical information, pathophysiology, and management of this condition. RE often occurs in individuals with normal development, and it is estimated that only a few new cases are identified each year in epilepsy centers. Approximately 10% of cases also occur in adolescents and adults. The hallmark feature of RE is drug-resistant focal seizures that can manifest as epilepsia partialis continua. Also, patients with RE usually develop motor and cognitive impairment throughout the years. Neuroimaging studies show progressive damage to the affected hemisphere, while histopathological examination reveals T-cell-dominated encephalitis with activated microglial cells and reactive astrogliosis. The current therapy guidelines suggest cerebral hemispherotomy is the most recommended treatment for seizures in RE, although significant neurological dysfunction can occur. Another option is pharmacological management with antiseizure medications and immunomodulatory agents. No significant progress has been made in understanding the pathophysiology of this condition in the last decades, especially regarding genetics. Notably, RE diagnosis still depends on the criteria established by Bien et al., and the accuracy can be limited and include genetically different individuals, leading to unexpected responses to management.

## 1. Introduction

Rasmussen encephalitis (RE) was first described by Sir Theodore Brown Rasmussen in 1958, a Canadian neurosurgeon who characterized RE as a chronic inflammatory neurological disease. Rasmussen encephalitis generally occurs in childhood and is characterized by recurrent seizures, delayed developmental milestones, progressive cognitive decline, neuroimaging with chronic inflammatory changes, and progressive hemispheric atrophy [1].

In many cases of RE, patients develop drug-resistant epilepsy and present neuro-logical deficits, which are commonly associated with typical radiological presentations of the disease [2]. Since the publication of the first case series about RE, a few hypotheses on the pathophysiology have emerged. Initially, Rasmussen proposed that a viral agent might be implicated in the etiology of this condition. However, several later studies extensively investigated viral agents possibly associated with RE, and none of these studies had positive results [2].

Histopathological studies have shown extensive T-cell infiltration in affected brain tissue correlating with the progression of the disease, as well as the genetic expression of multiple inflammatory cytokines. Nevertheless, a specific antigenic antigen that is a possible initial trigger is yet to be discovered. The unilateral presentation of the disease, with histopathological findings commonly isolated to one hemisphere of the brain, also remains a mystery, even though some studies have suggested that this unusual presentation might be related to a unilateral initiation of immune responses by an unknown antigen present in one of the brain hemispheres [2].

Antineuronal antibodies have also been detected in patients with RE, but this is likely an epiphenomenon instead of a causative event, considering the lack of response of RE patients to plasmapheresis. Microglial and astrocytic activation are also associated with RE, but further studies are needed. Therapeutically, the use of oral or intravenous steroids and intravenous immunoglobulins has led to a decrease in the frequency of breakthrough seizures. However, plasmapheresis, for example, has not led to significantly improved outcomes. Still, the only gold-standard treatment for this disorder is hemispherectomy, which, despite its side effects, has been proven effective in terms of clinical outcomes [3].

The current study aims to narratively review the literature about RE, including historical information, pathophysiology, and management of this condition. In the last decades, little progress has been made regarding the pathophysiology of RE. Also, the most used diagnostic criteria are still based on Bien et al.’s study, and its accuracy can be limited due genetically diverse individuals, leading to unexpected responses to management [4].

## 2. Natural History of Rasmussen’s Encephalitis

Rasmussen encephalitis (RE) is a persistent, localized encephalitis that gradually affects one hemisphere of the brain and has an unknown etiology. The syndrome generally first manifests between the ages of six and eight. Theodore Brown Rasmussen, a Canadian neurologist, first described it in 1958. After publishing a few case reports, he conducted a study with 27 children with chronic focal encephalitis characterized by a subclinical onset and relapsing seizures who were surgically operated and followed up from 1945 through 1976 [5,6,7,8,9]. Rasmussen originally characterized the syndrome as episodes of hemiparesis, cognitive impairment, and refractory focal motor seizures. Regarding the natural history of the condition, Rasmussen described: “(…) patients exhibited a slowly progressive hemiparesis, others had a stable, marked hemiparesis dating from an initial febrile convulsive episode [6]”.

The incidence of RE is estimated at 1.8 to 2.4 out of every 10 million people annually, or 0.18 per 100,000 people. This syndrome affects both sexes indiscriminately and with no known ethnic or geographic predisposition [10]. Interestingly, RE usually affects pediatric individuals with previously normal developmental milestones and a median age of 6 years, but around 10% of the individuals affected by RE are young adults [11]. Recurrent focal seizures are a characteristic clinical manifestation of RE, but patients can present with unilateral dystonia, athetosis, and limb paresis [12,13,14].

Seizures usually become frequent and resistant to medications. Also, individuals with RE may require recurrent hospital admissions for status epilepticus [15]. Patients with RE can have multiple seizure semiologies. Granata et al. revealed that 58% of patients developed multiple seizure types within three months of initial presentation [16]. Epilepsia partialis continua (EPC) is a rare seizure disorder with particular clinical associations, including RE. EPC may emerge at any point in the course of RE, with rates of 37 to 92%, within the first 3 to 5 years [17]. EPC is described in the International League Against Epilepsy (ILAE) (https://www.epilepsydiagnosis.org/, accessed on 2 April 2024) diagnostic manual as “recurrent focal motor seizures (typically affecting hand and face, although other body parts may be affected) that occur every few seconds minutes for extended periods (days or years)”. Some authors characterized EPC as a refractory focal somatomotor status epilepticus with continuous focal jerking of a body segment, typically localized to a distal limb or face and with cortical origin. Thomas et al. reported that EPC has a mean duration of 25 months and a range of 4 h to 18 years [18].

Initially, neurological deficits fluctuate with seizure burden but become permanent and may progress in severity. Notably, patients develop progressive functional deficits localizing to the affected hemisphere. Granata et al. found that 30% of the patients were wheelchair-bound within three years of the diagnosis [12]. Interestingly, individuals with left hemispheric predominance usually presented more significant impairment in verbal than non-verbal responses, whereas patients with right hemispheric disease had the opposite deficit. However, some authors affirmed that nonverbal performance declines over time regardless of disease laterality, which can be partially explained by epileptic activity, even though RE generally has unilateral pathological findings [19].

**Figure 1:**
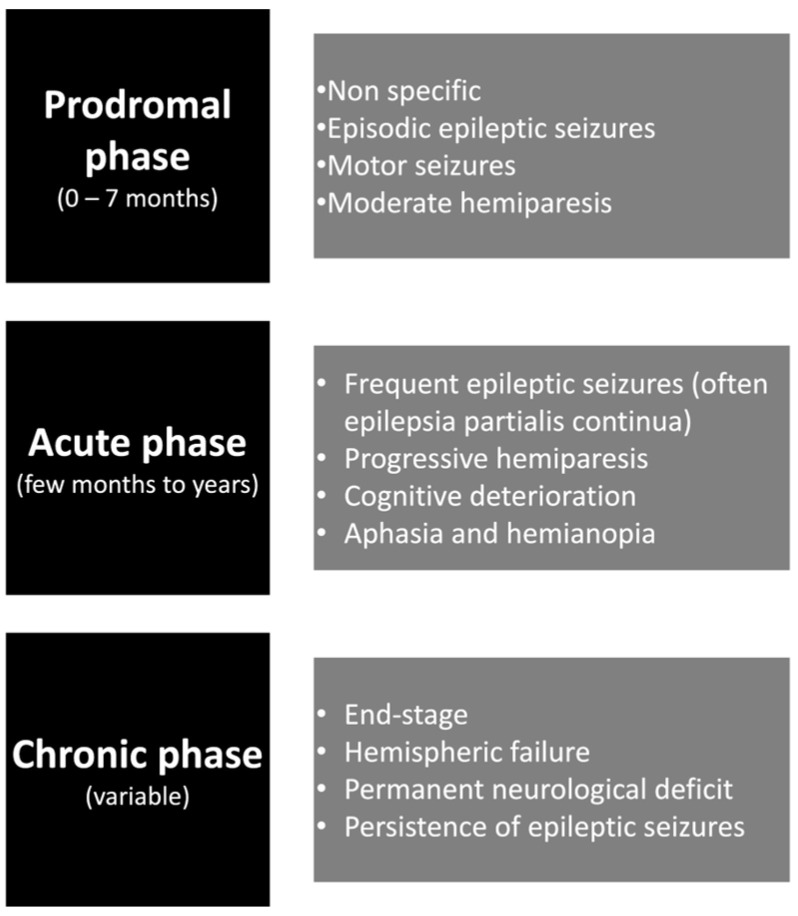
The natural history of Rasmussen’s encephalitis.

More recently, authors have divided the natural history of the disease into three stages: a prodromal stage, an acute stage, and a chronic (residual) stage (Figure 1). The first stage is characterized by mild hemiparesis and seizures, generally occurring in a low frequency. A few months later, RE individuals tend to exhibit a higher frequency of seizures, presenting as focal motor seizures or EPC. Unfortunately, if the appropriate treatment is not started during the acute stage, RE patients tend to develop worsening focal deficits, such as hemianopia, hemiplegia, behavioral changes, aphasia, and cognitive deficits. The “residual stage” is the last stage and is characterized by a decrease in the frequency of seizures and persistent neurological deficits [20].

## 3. Risk Factors

Some authors suggested that RE tends to occur in previously healthy individuals. However, a Canadian study described that 19% of the individuals with RE suffered from perinatal complications [21]. Fauser et al. reported that perinatal complications and autoimmune conditions are predisposing factors for the development of RE. Fever was found to be a trigger factor for RE in younger patients but not in adult patients [22]. Febrile illnesses usually occur within one month of RE onset and are related to an upper respiratory viral infection. It is suggested in several autoimmune disorders the role of molecular mimicry, bystander T cell activation, or viral persistence [23].

There are possible genetic factors associated with the development of RE. The as-sociation of RE with ipsilateral uveitis [24], membranous nephropathy [25], scleroderma [26], and Parry-Romberg syndrome [27,28] were already reported. Interestingly, in a study performed by Fauser et al., an increased frequency of twin pregnancy was seen in patients with RE. However, none of the twins were affected by RE [22]. The authors proposed that the uterine environment and the physiological conditions in twin pregnancy are related to the perinatal complications and increased risk factors for RE instead of purely genetic factors [22].

RE can occasionally be associated with other conditions, such as low-grade tumors and tuberous sclerosis. In one case, the association with an SCN1A mutation was re-ported [29]. Leitner et al. performed whole exome sequencing in the brain tissue of patients with RE, and they found several deleterious variants of unknown significance (ANGPTL7/MTOR, SCN1A, FCGR3B, MTOR) and common HLA variants in >25% of RE cases (HLA-DRB1, HLA-DQA2) [30].

## 4. Pathology and Pathophysiology

The pathophysiology of RE is not well-known. In one of his studies, Sir Theodore Brown Rasmussen described 27 individuals with a histology characteristic of active encephalitis, with large and small vessels showing perivascular lymphocytes and glial nodules scattered throughout the grey and white matter [31]. He further elucidated that there was a pattern to the microscopy, varying from patient to patient only in the degree of inflammatory changes and neurological sequelae. Dr. Rasmussen also described in detail the phases of pathogenic abnormalities seen in the patients: “(…) Destructive changes first appeared as laminar necrosis with inflammatory cells still prominent. As the destructive changes progressed to the stage of spongy degeneration, inflammatory cells were usually sparse or absent. In some patients, and some areas, there were inflammatory cells in the leptomeninges, but in most of the microscopic sections the leptomeninges showed little or no evidence of inflammatory activity, despite the presence of perivascular cuffing and glial nodules in the cortex beneath [8]”. He also hypothesized that a viral etiology could induce depression of antibody-mediated and lymphocyte-mediated immunity, leading to chronic encephalitis, similar to the process that occurs in subacute sclerosing panencephalitis. However, electron microscopic searches for viruses in those patients yielded negative results [8].

After the initial studies from Rasmussen, extensive neuropathological analysis of patients with RE has been carried out in search of several viruses, including Epstein-Barr virus [32], cytomegalovirus [33], herpes simplex virus [34], and enterovirus [35]. However, none of these studies was able to detect an association between viruses and RE. As Varadkar et al. mentioned, it is possible that infected cells under cytotoxic T-cell attack are damaged after being engulfed by microglia and macrophages, and detecting a virus would probably be easier at earlier phases of the disease, which is not the stage where most of the brain samples analyzed after neurosurgery are at, which does the search for a culprit pathogen even more challenging in RE [36].

A division into four groups has been proposed to help with the pathophysiology classification (Table 1). Group 2 has at least one gyral segment with full necrosis; Group 3 exhibits neuronal loss and gliosis; Group 4 exhibits gliosis and glial scarring. Group 1 exhibits inflammation along with microglial nodules [37]. The round cell infiltrates in RE brains consist almost exclusively of T lymphocytes [38]. Microscopic imaging might indicate a chronic encephalitis that is confined and has been simmering for years. Compared to other CNS inflammatory diseases, such as allergic encephalitis or post-infectious perivenous encephalitis, the lesion’s characteristics and location are more consistent with viral encephalitis, even though several microscopic viral studies have been negative in RE [6].

The affected hemisphere in RE has multiple foci progressing through different stages [39]. The lesions can be discrete and affect a large intralaminar portion of the cortex [39]. Still, the lesions may be bordered by normal central nervous system tissue, which can explain normal biopsy findings. Microscopically, T-lymphocytic infiltrate, reactive astrocytosis, activated microglia, and neuronophagia leading to neuronal loss and cortical atrophy are seen in brain parenchyma [39]. Most infiltrating lymphocytes in RE are cytotoxic T-cells, and long-lived clonal populations of cytotoxic T-cells were found in immunohistochemistry analysis of patients with RE [40]. Bernasconi et al. revealed that antibodies to a-amino-3-hydroxy-5-methyl-4-isoxazole propionic acid and NMDA receptors were found in 82% of patients with RE and 64% of patients with EPC [41]. However, the anti-glutamate receptor 3 (GluR3) is not always present in RE and is also not specific for this syndrome [41].

Given the histologic documentation of prominent T-cell involvement, RE is considered an immune-mediated disease. The activation of inflammatory pathways and release of cytokines such as IL-1 mediated by CD8+ T cells has also been shown in RE. It is also possible that oxidative stress damage and apoptosis are related to these pathways and play a role in microglial activation, neuronal loss, and the neurological deficits seen in RE. Gene expression studies have also shown the presence of additional cytokines and their receptors (CCL5, CXCL10, CCL22, CCL23, CXCL9, IFNγ, and Fas ligand) in RE [20]. Studies have shown a marked negative correlation between the expression of these cytokines (IFN-γ, CXCL5, CXCL9, CXCL10) and the period from the onset of seizure to surgery [42]. Owens et al. reported that T cells isolated from RE brain surgery specimens in the early stages of the disease expressed CD103 [43]. Resident memory T cells express CD103 and/or CD69, which reside in the affected tissue even after the pathogen is cleared and do not circulate in the bloodstream [44].

Although studies have demonstrated a Th1 immunological response involving both CD4+ and CD8+ cells in RE, no association has been found with a specific antigen that might have initially triggered those immune and inflammatory reactions [20]. Ramaswamy et al. have also demonstrated an increased expression of inflammation related genes in RE, such as IL-1beta, IL18, CASP1, NLRP1, and NLRP3 [45]. As Varadkar et al. pointed out, the unilateral involvement of the CNS in RE may be caused by inappropriate activation of the immune system in those who are genetically predisposed due to an infectious trigger localized in one hemisphere or against self-antigens that are not equally expressed in both hemispheres [36].

Liba et al. revealed that patients with RE have increased CD8+ T cell subpopulation in cerebrospinal fluid (CSF) and its reduction in blood and persistently increased levels of C-X-C motif ligands (CXCL10 and CXCL13) and B-cell activating factor (BAFF) in CSF compared with the controls [46]. Interestingly, these biomarkers tended to decrease during therapy but remained above the 95th percentile compared to controls [46]. Also, the C-X-C motif ligands were associated with the maintenance of intrathecal inflammation in some types of encephalitis [47]. Interestingly, Thom et al. reported HIV in the absence of opportunistic infection or vasculitis, presenting with seizures, severe cortical atrophy, and CD8 cortical infiltrates resembling RE [48].

Pechlivanidou et al. reported a case of RE associated with antibodies to a subunit of one of the major neuronal nicotinic acetylcholine receptor subtypes (α4β2) [49]. Also, Spitz et al. reported autoantibodies directed against voltage-gated potassium channels [50]. Bien et al. (2005) proposed that the positivity of antibodies against glutamic acid decarboxylase (anti-GAD) should be considered in the differential diagnosis [4]. On the other hand, some authors reported cases of RE associated with anti-GAD [51].

Additionally, humoral response has also been studied in RE. Antibodies such as anti-GluR3, mammalian uncoordinated (Munc)-18−1, the alpha-7 nicotinic acetylcholine receptor, and the GluR2-GluR3 subunit complex of the al-pha-amino-3-hydroxy-5-methyl-isoxazole-4-propionic acid (AMPA) receptor have already been identified in the brain of RE patients [52]. Some studies have identified anti-GluR3 antibodies in other types of epilepsy, and other anti-neuronal antibodies, such as Munc-18 and the alpha7-acetylcholine receptor, were identified in the serum from a few patients with RE [52]. Also, antibodies against delta 2 and epsilon 2 in GluR2 were already found in individuals with meningoencephalitis [53]. Leucin-rich glioma inactivated 1 (LGI1), α-amino-3-hydroxy-5-methyl-4-isoxazoleproprionic acid (AMPA) or gamma-aminobutyric acid (GABA) receptors antibodies, and have also been identified in limbic encephalitis which is a condition characterized by seizures, and patients improved after being treated with immunotherapy [36].

Neural autoantibodies binding to synaptic agents have already been identified in patients with drug-resistant epilepsy, and a case of anti-N-methyl-d-aspartate (NMDA) encephalitis mimicking RE has also been reported, even though NMDA antibodies are infrequent in RE [42]. Greiner et al. reported unilateral frontal lobe abnormalities on MRI and FDG-PET in a case of NMDA encephalitis [54]. Gurcharran et al. reported the ambiguity of anti-NMDA receptor antibodies, which likely cause and the consequence of the pathogenic process [55]. However, the clinical significance of those antibodies is yet to be determined, especially because they have been found bilaterally in the brains of RE individuals, which does not coincide with the disease’s histological pattern. The fact that the appearance of these antibodies might be a consequence of the pathological process of RE and not be directly associated with the etiopathogenesis of the disease in itself might explain why B-targeted treatments and plasmapheresis have a low efficacy on the disease, for example [55].

As for microglial-mediated neurodegeneration, increased pannexin hemichannels have been hypothesized to be implicated in RE epileptogenesis. Additionally, upregulated endosomal Toll-like receptors (TLRs) in microglia increase the brain’s susceptibility to infiltration of T cells, increasing the activation of inflammatory pathways. It is also relevant to consider the role of the activation of astrocytes in RE because since this mechanism has been demonstrated throughout cortical damage and because astrocytes are essential in triggering and maintaining inflammation during epileptogenesis, it is possible that activated astrocytes are also involved in the epileptogenic process of RE. Previous studies have also shown the binding of endogenous high-mobility group box-1 (HMGB1) to TLRs in the cytoplasm of activated astrocytes in RE specimens, which could indicate that an HMGB1-TLR pro-inflammation pathway in astrocytes might represent a potential target [42].

Few studies have investigated the role of genetic factors in RE. Case reports have described mutations in the NOD2/CARD15 and SCN1A genes [29,56]. Also, single nucleotide polymorphisms in the CTLA4 and PDCD1 genes have also been described [57]. In this context, Junhong Ai et al. investigated genetic factors of RE performing whole exome sequencing (WES) in 15 RE patients. Interestingly, they found genes with single nucleotide variants (SNVs) responsible for antigen presentation (HLA-DQA1, HLA-DQB1, HLA-DRB5, and CD1A) and antiviral infections (TRIM41). The authors also found three genes related to epilepsy (ADGRV1), schizophrenia (CMYA5), and nerve cell regeneration (TNR) [58].

It is worth mentioning that in the earlier phase of the disease, hemispherotomy is usually the most indicated therapy, and that in the acute and later stages of the disease where surgery is often contraindicated due to increased risk of functional sequelae, immunotherapies are usually recommended. Immunotherapies can also be beneficial in the prodromal stages of the disease and could halt the early inflammatory processes, however immunotherapies should not delay surgery [59].

## 5. Neuroimaging

Brain MRI is useful to confirm the suspicion of RE, even though it can be normal at the beginning of the disease [60]. Unilateral localized cortical edema may be observed initially. In the early stages, T2-weighted and fluid-attenuated inversion recovery (FLAIR) hyperintensities gradually appear in the affected side’s cortex and subcortical white matter. As the illness advances into chronic phases, unilateral atrophy of the brain and basal ganglia may be seen. Hyperintensities may disappear in the latter stage, resulting in a noticeable hemi cerebral atrophy. Also, some authors proposed that the extent of the high-intensity lesion did not correlate with the frequency of seizures, but the sequential changes in MRI seemed to reflect the course of this disease [61].

Bien et al. proposed phases mainly consisting of T2-weighted sequences based on a retrospective analysis of ten patients’ first and follow-up MRIs (Table 2). The pattern of atrophy is not uniform, and several cortical patterns have been reported [62]. Apparently, the frontal lobe and insula volume loss are the most frequently noticed abnormalities [63]. On the other hand, alterations in the posterior lobes are less commonly observed in the early stages of RE [1]. Historical studies reported that the atrophy process predominantly involved the basal ganglia among the subcortical structures, but more recent neuroimaging studies found the most pronounced atrophy in the temporomesial region [1]. Interestingly, some patients with RE can flatten the caudate head, changing the frontal horn contour, a landmark finding that suggests RE [64].

Additional tests, such as magnetic resonance spectroscopy (MRS), 18 F-fluorodeoxyglucose positron emission tomography, and Tc 99m HMPAO single photon emission computed tomography [65], can verify the unilateral features of suspected RE. Functional abnormalities may occur early in the disease course, preceding structural changes. The functional tests assess regions of hypoperfusion and hypometabolism concerning RE. Sometimes, hyperfunction can be observed in individuals with active epileptic foci. Interestingly, as in structural changes, the changes in the metabolism usually occur in the anterior lobes [66]. In cases of MRS, decreased levels of N-acetylaspartate and creatine, suggesting neuronal death, and increased levels of choline, glutamine, and glutamate, suggesting demyelination, can be observed [60]. Also, magnetoencephalography can be used to identify the localization source of the seizure [67].

Wang et al. proposed to apply automated quantitative volumetric MR analysis to patients with suspected RE [68]. They found that interhemispheric and frontal lobe ratios had the best prediction in the diagnosis of RE at the early stage of the disease [68]. Also, the authors observed that the volumetric analysis can also be used to monitor disease progression by calculating the decrease in volume in the hemispheric and lobar regions [68]. In this context, the global volumetric loss is 2.8 to 5% per year in the mainly affected hemisphere and 0.2 to 1.1% per year in the contralateral [1,69]. It is worth mentioning that a compensatory brain volume increase in the contralateral hemisphere has already been reported [70].

Bauer et al. investigate subcortical grey matter volumes and asymmetries in RE. Also, the individuals were classified as type 1 (onset ≤ 6 years) or type 2 (onset > 6 years). They found that unihemispheric cortical degeneration was accompanied by ipsilesional atrophy of the nucleus accumbens, caudate nucleus, putamen, thalamus, and contralesional atrophy of the nucleus accumbens and caudate nucleus both in type 1 and type 2. In type 1, however, a contralesional volume increase of the amygdala, hippocampus, pallidum, and thalamus was found. Both ipsilesional and contralesional subcortical atrophies, like cortical atrophy, are most probably caused by neurodegeneration following chronic neuroinflammation. They speculated that contralesional volume increase in type 1 could be related to neuroplasticity or ongoing acute neuroinflammation [71].

## 6. Electroencephalography

Electroencephalography (EEG) is important in the early stages of the disease even though surface EEG is generally not sensitive or specific enough for localization of the epileptic foci [4]. Also, EEG should be performed after an acute change in the neurological examination of patients with RE. However, an association has been demonstrated be-tween EEG results and the course of the disease. For example, when epilepsy first begins, the EEG is frequently normal at first, but months later, chronic high-amplitude activity appears in the affected regions. Background slowing activity may also be associated with epileptiform abnormalities. The first EEG change is persistent focal slowing, which progresses to slowing involving one or both hemispheres with marked asymmetry [17]. Within six months (in 25% of patients), further interictal anomalies in the non-atrophied hemisphere may manifest in certain situations [17]. Although these contralateral EEG abnormalities may indicate cognitive deterioration, they do not appear to suggest bilateral illness [36]. Based on these findings, Longaretti et al. proposed that RE is an epileptic encephalopathy [17], which is defined by the ILAE as “a condition in which the epileptiform abnormalities themselves are believed to contribute to the progressive disturbance in cerebral function [17]”.

Ictal recordings of EPC do not correlate with EEG 43% of the time, and recurrent subclinical seizures are observed in 42% of patients with RE [17]. Also, in half of the individuals with RE at 6 months of disease, epileptiform discharges will be present more than seventy percent of the time [17].

## 7. Diagnosis

There are still no biological markers to provide a definitive diagnosis of RE. The presence of oligoclonal bands, elevated protein levels, and hypercellularity of cerebrospinal fluid (CSF) were already proposed, but less than half of the individuals with RE present these findings [4]. These criteria aim to reach an early diagnosis and eventually limit the progression of cerebral atrophy (Table 3).

Nevertheless, atypical presentations of RE remain undiagnosed with Bien et al. proposed criteria [4]. Interestingly, bilateral RE cannot be diagnosed through these criteria [20]. In this context, Olson et al. [72] reviewed the criteria proposed by Bien et al. [4] to increase their sensitivity. They explained that specificity is not a concern for RE diagnosis criteria because false positives had an identifiable alternate diagnosis. Also, the authors recommended diagnosing RE in the absence of EPC, without progressive cortical deficits, or in the presence of a normal brain MRI. In this way, Olson et al. suggested considering patients meeting criteria B3 plus two of three A criteria as positive and expanding the pathology criteria to allow for stages of RE [72]. For a critical analysis of RE cases with missing or atypical features from the Olson et al. cohort [72], see Table 4.

Korn-Lubetzki et al. reported two cases of biopsy concerning RE with active inflammation and delayed seizure onset. The authors stated that seizures are not an obligatory presenting symptom of RE [73].

Patients presenting in the early stages of RE without EPC are challenging to diagnose. This is more evident in pediatric individuals. Some of the neuroimaging abnormalities can be under-interpreted as normal variants. Also, the fact that unilateral cortical deficits without EPC can be diagnostic is worrisome because it could lead to the misleading overdiagnosis of RE. Biomarkers are needed for diagnostic clarification of RE; otherwise, several individuals may be misdiagnosed.

## 8. Pharmacological Management

Anti-seizure medications (ASM) have limited efficacy in RE, especially because the RE pathophysiology is associated with chronic inflammation, resulting in drug-resistant epilepsy and progressive destruction of the cerebral hemisphere. Thus, treatments targeting the immune system are recommended, especially in the early stages of the disease, in patients with slow disease progression, mild deficits, and not eligible for surgery [20].

There is no ASM therapy, mono- or polytherapy, showing superiority in the management of seizures in patients with RE. ASM should be used to limit the number of seizures and prevent seizure recurrence, as well as status epilepticus. EPC is particularly unresponsive to ASM. Thus, it is generally recommended that any ASM be chosen based on the empirical demonstration of efficacy and tolerability in every patient. Also, it is not recommended to use any high-dose combination of ASM to limit side effects that can be related to the polytherapy administration [20].

**Figure 2:**
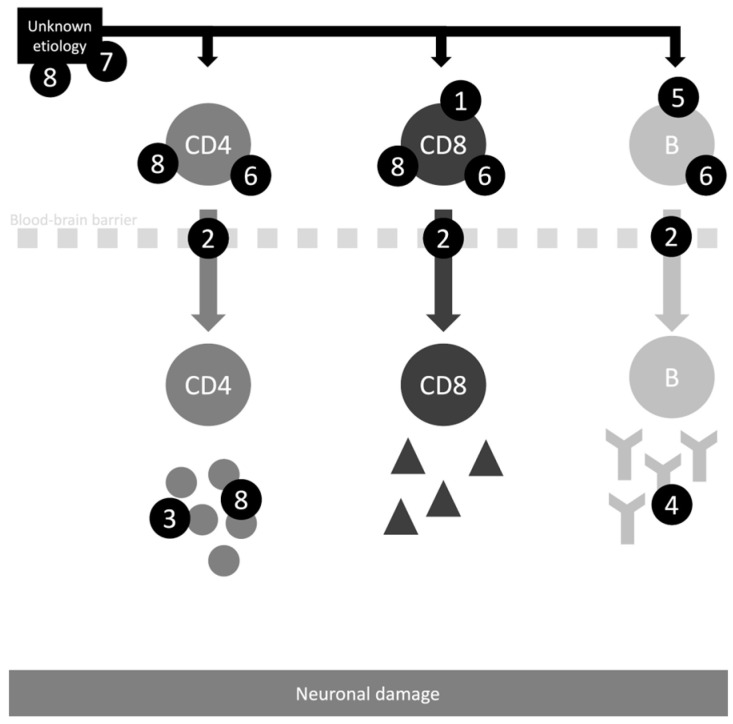
Schematic diagram of the proposed mechanisms for the drugs related to Rasmussen’s encephalitis management. 1—Inhibition of lymphocyte T proliferation (tacrolimus, mycophenolate mofetil, and azathioprine). 2—Diapedesis blockage (natalizumab). 3—Pro-inflammatory cytokines blockage (adalimumab, anakinra, and infliximab). 4—Antibody blockage/ clearance (intravenous immunoglobulin, plasmapheresis, and adsorption). 5—Inhibition of lymphocyte B proliferation (rituximab). 6—Inhibition of T and B lymphocytes (alemtuzumab, mitoxantrone, cyclophosphamide). 7—Unknown etiology (ganciclovir). 8—Broad-spectrum anti-inflammatory action (corticosteroids).

For further understanding of the current therapy and related pathophysiology, see Figure 2.

It is worth mentioning that some immunotherapies showed efficacy in reducing seizure frequency, and some even reduced EPCs. On the other hand, some immuno-therapies were not effective in reducing seizures, but they slowed the functional decline in motor and language functions in individuals with RE. For a detailed analysis of the different medical therapies already attempted to manage RE, see Table 5.

On some occasions, immunotherapy will likely have better outcomes than surgery. The acute and diffuse inflammatory response significantly affects the prodromal stage in pediatric individuals. In this context, immunotherapy should be tried before extensive neurosurgical procedures. Also, immunotherapy is recommended for patients with significant cardiovascular risk or poor post-surgery prognosis with limited neurological deficits. Another possible recommendation is that immunotherapy function as a bridging therapy until surgery [123].

Corticosteroids are effective first-line medications or in seizure aggravation cases in RE, but they should be followed by another long-term medication due to their significant side effects. Because corticosteroids act in the innate and adaptive immune system, studies have shown decreased expression of pro-inflammatory genes (IL-1, IL-2, IL-6, TNF-alpha, IL-2R, and adhesion molecules) following corticosteroid administration [79].

Other studies have reported using immunoglobulin (IVIg) as first-line or add-on therapy in RE. IVIg seems to have an immunomodulatory effect on the characteristic T cell-induced damage of RE through the inhibition of multiple co-stimulatory pathways [20]. There are three main hypotheses for explaining the mechanism of IVIg effectiveness in RE. First, IVIg is believed to have anti-idiotype antibodies that could sequester au-to-reactive antibodies related to RE. Second, IVIg can dilute the concentration of au-to-reactive antibodies, preventing binding these antibodies to the Fc receptors. Third, IVIg can saturate the Fc receptor and competitively inhibit autoreactive antibodies [124].

In a randomized prospective therapy study, 16 individuals with recent onset RE were randomized to intravenous IVIg or tacrolimus. Despite neither treatment being more effective, those who received immunotherapy survived longer than those who did not [10]. According to a study that compared immunotherapy with surgery, one out of eleven individuals who received immunotherapy became seizure-free, whereas nine out of ten who underwent surgery achieved seizure remission [125]. Caraballo et al. reported that the combination of steroids and IVIg is associated with an improvement in seizure frequency, EPC disappearance, and stabilization of neurological deterioration [126]. Also, a number of conditions in addition to RE are associated with poor response to the first-line therapy [127]. Interestingly, IVIg in cases of focal epilepsy with positive anti-GluR3 antibodies was not effective [128]. However, in syndromes with EPC due to multifocal encephalitis, IVIg was effective in ceasing seizures [129].

Plasmapheresis and immunoadsorption were studied because many patients with RE have autoantibodies (anti-GluR3 antibodies). These treatments decrease circulating antibodies and other soluble immune mediators [20]. However, plasmapheresis had a low effective rate of reducing the seizure frequency and short-timed efficacy with recurrence of seizure burden, probably due to the lack of specific T-cell-induced cellular damage. Therefore, some authors proposed that corticosteroids should be first-line therapies, and immunoglobulins should be reserved for corticosteroid failure or adjunctive treatment with corticosteroids before changing to another therapy. Also, plasma-pheresis should be attempted if the concurrent treatment of corticosteroids and IVIgs fails.

Tacrolimus was one of the first medications targeting lymphocytes in individuals with RE. The use of tacrolimus should be limited for patients with low seizure burden and significant neurological impairment. Tacrolimus is a calcineurin inhibitor that is active in the T-cell immune response through the inhibition of IL-2 production. Previous studies have suggested that tacrolimus should be used as a single agent and in the transition of treatments after using corticosteroids. However, in a drug trial, tacrolimus showed a positive effect in slowing the progression of the disease without a significant effect on seizures. The clinical response was similar to that of IVIG overall, but tacrolimus presented an increased percentage of adverse events [20]. On the other hand, azathioprine in RE showed a significant effect on seizure burden but limited efficacy in disease progression. The data for specific recommendations for mycophenolate mofetil and rituximab are scarce.

Targeting pro-inflammatory cytokines was believed to be effective after a patient with RE and granulomatosis showed improvement with infliximab [56]. In this context, medications targeting tumor necrosis factor-alpha (TNF-α), like adalimumab, have been studied in RE. Lagarde et al. proposed that adalimumab should be reserved for patients with RE who have a slow progression and those who are not surgical candidates [111]. A clinical trial with anti-TNF-α medications in RE is currently being performed (https://clinicaltrials.gov/ct2/show/NCT04003922, accessed on 24 February 2024). The data is scarce regarding interleukins, especially anakinra, a modified recombinant version of the human interleukin one receptor antagonist (IL-1Ra) protein. Therefore, anti-TNFα and IL-1Ra should be studied because they can potentially mitigate seizures and inflammation related to RE.

Natalizumab showed significant efficacy in managing mouse models of RE, but the same efficacy was not observed in humans [115]. It is believed that natalizumab has a more pronounced efficacy during the early stages of RE. Also, some authors observed that patients not responsive to natalizumab had lower levels of inflammatory cytokines and T-cell infiltration in the brain tissue [46].

Cyclophosphamide only showed transitory efficacy, and its significant side effects, including cumulative toxicity, do not contribute to its use in RE. Mitoxantrone showed similar findings to cyclophosphamide, limiting its recommendations. Other medications with single-case or case-series reports and limited data were acyclovir [118], ganciclovir [119], zidovudine [113], thalidomide [121], cyclosporins [110], basiliximab [115], stem cell transplantation [115], intrathecal infusion of autologous adipose-derived regenerative cells [130], and intraventricular interferon alfa [110,131,132,133].

**Figure 3:**
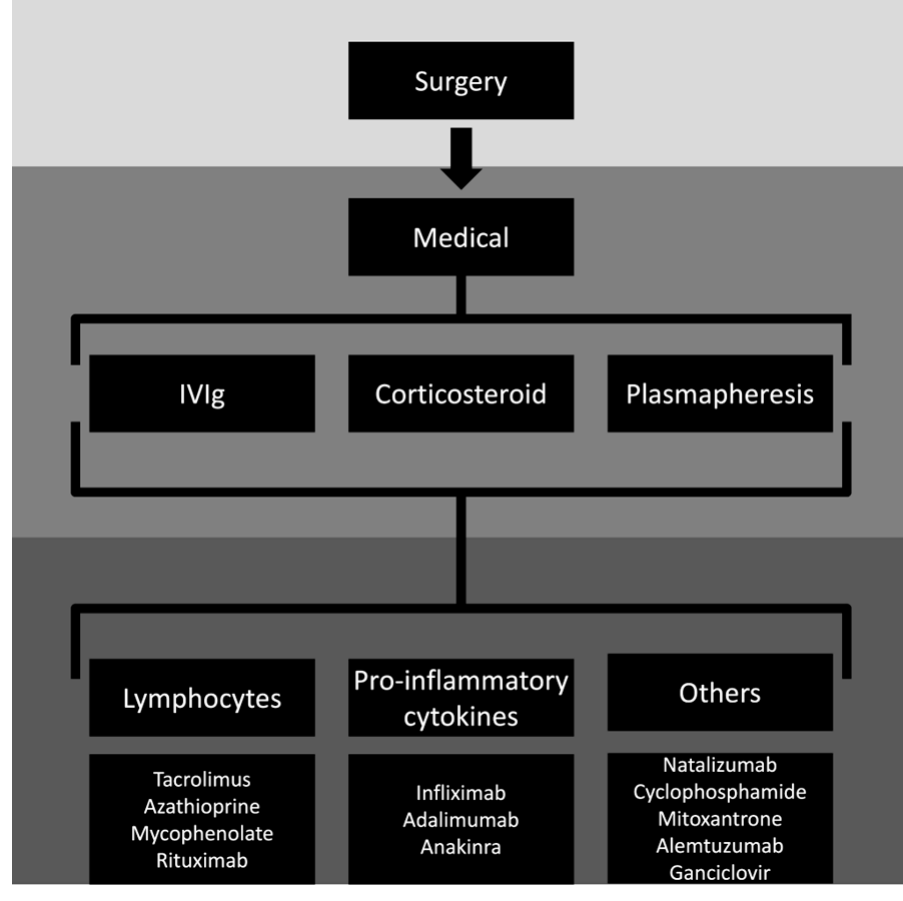
Management of Rasmussen’s encephalitis. First, surgery eligibility should be assessed. Second, medical treatment can be used independent of the indication of the surgical procedure. The first-line therapies are corticosteroids ± intravenous immunoglobulin (IVIg) ± plasmapheresis/immunoadsorption. Second-line therapies are related to targeting lymphocytes, pro-inflammatory cytokines, and other mechanisms. The data is scarce in providing specific definitions for most second-line therapies in RE.

Follow-up of hemiparesis [21], cognitive performance in individuals without hemi-paresis [134] and hemispheric ratio on neuroimaging can all be used to monitor clinical progress in these patients. The hemispheric ratio is the affected/unaffected hemisphere ratio calculated using axial and coronal slice planimetry, incorporating the Sylvian fissure. Most tissue loss happens within one year of the acute stage’s beginning [62]. For a summary of the management of RE, see Figure 3.

## 9. Neurosurgical Management

For drug-resistant epilepsy associated with RE, surgery is still the most effective treatment. Several surgical techniques have been developed in the past century. In the early 1950s, hemispherectomy was a popular technique and involved removal of the affected cerebral hemisphere. However, because of a high rate of complications such as chronic subdural fluid collection, hydrocephalus, and severe neurological sequelae, less invasive technical variations were developed, such as hemispherotomy, which consists of less resection, leaving intact the live functionally disconnected cerebral hemisphere [135].

The best time to perform surgery in patients with RE is controversial. Some authors proposed that it should be done early in the disease course to prevent the spreading of the abnormalities to the other hemisphere, halting seizure relapse and possibly cognitive decline with the progression of the disease [36]. Noteworthy, focal resective surgeries can be effective in adults and adolescents due to the usually localized disease [136].

For most specialized centers in RE, the most common neurosurgical procedures are subtypes of hemidisconnection since they have minimal blood loss and surgical risks. Some hemidisconnection techniques are the modified functional hemispherectomy, the peri-insular hemispherotomy, the parasagittal hemispherotomy, and the endoscopically assisted hemispherotomy [137]. Notably, these procedures require technical experience, and there is an increased risk of failure due to incomplete disconnection compared to hemispherectomy techniques.

The risk of adverse events related to neurosurgical procedures should be evaluated case-by-case. Most patients with RE already have some degree of hemi-sided weakness, so the main risk for these patients would have been a certain increase in spastic hemiparesis and, of course, new hemianopia. So, the main question for the subjects and caregivers is the benefits of seizure freedom in overcoming these side effects. Noteworthy, seizures have a significant impact on the quality of life.

Bellamkonda et al. reported a cohort of 44 patients with RE who underwent epilepsy surgery. The authors described that anatomical hemispherectomy, compared to functional hemispherectomy, was independently associated with a longer time to postoperative seizure recurrence (HR 0.078, *p* = 0.03). There was no statistically significant difference in postoperative seizure recurrence between patients with complete hemispherectomy and those who had less-than-hemispheric surgery. Following surgery, 68% of the patients could ambulate, and 84% could speak regardless of operative intervention. Another interesting finding is that 40% of the patients will have postoperative seizures [138].

Schramm et al. reported that functional outcomes after adult HS for patients with RE have good quality of life scores and functional outcomes. The quality of life improved in 85% of the individuals, and 80% of the individuals were seizure-free [139]. Harris et al. found that most pediatric patients undergoing resection or hemispheric surgery for RE achieve good seizure outcomes [140]. For a detailed analysis of the different surgical therapies to manage RE, see Table 6.

### Cognitive Outcomes After Surgery

Surgical disconnection techniques are associated with side effects, and the most frequently reported are hemiparesis, hemianopia, and aphasia. Post-operative rehabilitation is essential for good outcomes regarding motor and language impairment. Fine motor control, language, and cognitive outcomes have a reserved prognosis. Patients can show a significant improvement in their gait with device assistance. Also, they typically show compensation for the visual field cut [11].

Borne et al. assessed the cognitive reorganization and outcomes in individuals with RE undergoing HS [162]. The authors showed that language and other cognitive functions usually improve after HS despite the permanence of deficits and neurological sequelae. Furthermore, language may remain a domain of the right hemisphere after left HS, despite reduced brain plasticity in late childhood and after left lateralization; nonetheless, deficits are generally significant. Additionally, even though the language is not as compromised after right HS as left HS, deficits can still be noticed [162]. To further assess the studies in the literature about cognitive outcomes after HS in individuals with RE, consider reading Table 7.

## 10. Differential Diagnosis

Even though MRI abnormalities in RE tend to progress in a characteristic manner, with initial phases marked by edema and high signal on T2 sequences in the affected hemisphere and subsequent atrophy and disappearance of the abnormal sign, bilateral presentation can also occur as mentioned above, which could lead to diagnostic delay and belated treatment. Moreover, the pathological findings in RE are nonspecific and could mimic focal viral encephalitis [36].

Derry et al. published a case of unihemispheric cerebral vasculitis mimicking RE. They pointed out that, unlike RE, there is no typical clinical presentation of cerebral vasculitis. Focal, multifocal, and disseminated disease presentations of cerebral vasculitis are all described. In their case, the brain MRI findings showed progressive unilateral cerebral atrophy resembling RE. However, the intracranial calcification present would be atypical. With the unusual presentation in this case, histological diagnosis was necessary and revealed a heterogeneous inflammatory infiltrate of the blood vessel walls pre-dominantly by lymphocytes. This was in contrast with the histologic abnormalities in RE, which tend to be more widespread, characterized by pan-encephalitis with aggregates of microglial cells and lymphocytes in the cortex, neuronophagia, and, at the end stage, widespread neuronal loss. In both biopsy specimens, the inflammatory process was restricted to the vessels [197].

For further reading about the possible differential diagnoses of RE, consider reading Table 8.

### Differential Diagnosis of Cerebral Hemiatrophy

Cerebral hemiatrophy, or unilateral brain atrophy, is the end-stage of various pathologies that culminate in the atrophy or hypoplasia of a single cerebral hemisphere. There are few potential differential diagnoses, especially when only pediatric individuals are evaluated. For further differential diagnoses of cerebral hemi-atrophy syndromes, read Table 9. There are no particular anomalies that set Rasmussen’s encephalitis apart from other etiologies of focal epilepsy. Findings from MRIs and clinical examinations are very helpful in distinguishing other pathologies from RE. Other conditions that can be presented with cerebral hemiatrophy include Sturge-Weber syndrome, hemi-megalecephaly, and Dyke-Davidoff-Masson syndrome, which are all typically linked to hemiplegia and epilepsy. Since RE is often resistant to antiseizure medications, their effectiveness in treating the disease may be limited.

## 11. Future Studies

It remains a mystery why RE pathophysiology involves only one hemisphere’s cortical and subcortical structures and why it is more likely to occur in early childhood. Although several hypotheses have been proposed regarding RE pathogenesis, further studies need to be conducted, especially regarding genetics and identifying a possible antigen that may initially trigger the immune response leading to inflammation, epileptogenesis, neuronal loss, and neurological sequelae. It is also relevant to conduct investigations on the humoral activation suggested in RE to identify if new antibodies or the previously identified molecules are part of an epiphenomenon or are related to the etiology of the disease, as well as the role of microglial and astrocyte activation.

An important matter to be addressed by future studies is the drug-resistant epilepsy in these patients, which causes a profound negative impact on patients and their families. Novel neurosurgical approaches should be tried to decrease disabilities and improve patients’ quality of life with RE. The variable clinical courses of the condition, with some individuals responding to pharmacological therapy while most require surgery, also require further investigation. It is imperative that clinical trials and experimental neuropathological studies are executed in the future to elucidate the pathophysiology of this condition and discover new drug targets.

Modifications of the current diagnosis criteria of RE are warranted in the following years. The current proposed diagnosis has several mimickers, which can lead to a mixture of different conditions with the same diagnosis. This can partially explain the different responses to the variety of therapies already studied in patients with RE. Studies of new biomarkers are needed to improve diagnostic accuracy.

## 12. Conclusions

Rasmussen encephalitis (RE) is a rare, progressive, chronic encephalitis that usually affects only one brain hemisphere. This disease is characterized by focal seizures, with motor and cognitive deterioration, and occurs mainly in children. Neuroimaging investigation with brain MRI and EEG can support the diagnosis. The management is medical (to reduce seizure severity and frequency) and surgical. The prognosis of the patient with Rasmussen encephalitis widely varies.

## Figures and Tables

**Table 1:** Histological classification proposed by Robitaille et al. (1991) [37].

Group	Description ^I^
Group 1	Inflammation with numerous microglial nodules, with or without neuronophagia, perivascular round cells, and glial scarring.
Group 2	Several microglial nodules, cuffs of perivascular round cells, and at least one gyral segment of complete necrosis
Group 3	Neuronal loss and gliosis with moderately abundant perivascular round cells and few microglial nodules.
Group 4	Few microglial nodules, neuronal loss, and mild perivascular inflammation, combined with various degrees of gliosis and glial scarring

^I^ Robitaille et al. performed standard histochemical staining techniques.

**Table 2:** Neuroimaging stages of Rasmussen’s encephalitis proposed by Bien et al. (2005) [4].

Stage	T2/FLAIR ^I^	Brain Volume ^I^
0	No abnormality	No abnormality
1	Hyperintense	Swelling
2	Hyperintense	Normal
3	Hyperintense signal	Atrophy
4	Normal signal	Atrophy (usually progressive)

^I^ The study protocol was based on brain magnetic resonance imaging assessed with T2-weighted (T2) and fluid-attenuated inversion recovery (FLAIR) sequences. The signal was defined as either normal or increased. Volume was characterized as normal, increased (cerebral swelling), or reduced (atrophy).

**Table 3:** Clinical criteria for Rasmussen encephalitis, adapted from Bien et al. (2005) [4].

Part A (All 3 Criteria Need to Be Marked) ^I^	
1. Clinical	Focal seizures (±epilepsia partialis continua) AND unilateral cortical deficit(s)
2. EEG	Unihemispheric slowing ± epileptiform activity AND unilateral focal seizure onset
3. MRI	Grey or white matter T2/FLAIR hyperintense signal or atrophy of ipsilateral caudate head
**Part B (2 of 3 Criteria Need to Be Marked) ^I^**	
1. Clinical	EPC or progressive ^II^ unilateral cortical deficit(s)
2. MRI	Progressive ^II^ unihemispheric focal cortical atrophy
3. Histopathology	T-cell dominated encephalitis with activated microglial cells (cerebral biopsy) and reactive astrogliosis ^III^

Abbreviations: EEG, electroencephalogram; EPC, epilepsia partialis continua; FLAIR, fluid-attenuated inversion recovery; MRI, magnetic resonance imaging; ^I^ If the criteria from Part A are not fulfilled, check the criteria in Part B. ^II^ Progressive implies at least two sequential examinations or studies. ^III^ Numerous parenchymal macrophages, B-cells, plasma cells, or viral inclusion bodies exclude the diagnosis of RE.

**Table 4:** Critical analysis of cases described by Olson et al. (2013) [72].

False-Negative Cases		
Criteria Achieved	Criteria Lacking	Missing or Atypical Feature
A1, A2, B3	A3, B1, B2	Normal MRI; lack of EPC or progressive cortical deficits
A1, A2, B3	A3, B1, B2	Bilateral EEG slowing and epileptiform activity, and bilateral seizure onsets; lack of progressive atrophy, progressive cortical deficits, or EPC
A1, A2, B3	A3, B1, B2	Mild atrophy on MRI but not meeting A3 criteria and without progression; lack of progressive cortical deficit or EPC
B3	A1, A2, A3, B1, B2	Lack of cortical atrophy, lack of focal cortical deficit, lack of unilateral slowing on EEG, lack of EPC
Atypical features in cases of RE satisfying the Bien clinical criteria and with consistent biopsies		
A1, A2, A3, B1, B2, B3	None	Hemi-choreoathetosis
A1, A2, A3, B1, B2, B3	None	Slowly progressive course
A1, A2, B1, B3	A3, B2	MRI with signal change without atrophy
A1, A2, A3, B1, B2, B3	None	MRI with ipsilateral or contralateral cerebellar atrophy
A1, A3, B1, B2, B3	A2	EEG with bilateral slowing and epileptiform activity
A3, B2, B3	A1, A2, B1	EEG with generalized epileptiform activity and generalized seizures
A1, A2, A3, B1, B2	B3	Dual pathology with cortical dysplasia and RE
A3, B2, B3	A1, A2, B1	Seizure semiology with generalized seizure
A3, B2, B3	A1, A2, B1	No seizures observed

Abbreviations: EEG, electroencephalogram; EPC, epilepsia partialis continua; MRI, magnetic resonance imaging.

**Table 5:** Medical treatment of Rasmussen’s encephalitis.

Reference	N ^I^	AAO ^II^	Protocol	Seizure Efficacy ^III^	Neurologic Symptoms Efficacy ^IV^	Note
Corticosteroids						
Dulac et al. (1991) [74]	5	NA	IV MTP 400 mg/m^2^; PO PDN 2 mg/kg	3/5 (60%) IMP	1/5 (20%) motor IMP	NA
Chinchilla et al. (1994) [75]	8	5	IV MTP 400 mg/m^2^; PO PDN 2 mg/kg	5/8 (62%) EPC ceased; 3/8 (37%) seizure IMP	5/8 (62%) hemiparesis resolved	AE: Cushing syndrome, infection, hypertension, and osteoporosis
Hart et al. (1994) [76]	10	5.5	IV MTP 400 mg/m^2^; PO PDN 2 mg/kg	5/10 (50%) seizure IMP	NA	NA
Krauss et al. (1996) [77]	1	15	PO PDN 50 mg/d	IMP	Cognitive IMP	NA
Granata (B) et al. (2003) [12]	14	4.9	IV MTP 20 mg/kg/d; PO PDN 1 mg/kg/d; ACTH	11/14 (78%) IMP	2/14 (14%) neurological IMP	NA
Bahi-Buisson et al. (2007) [78]	11	6	IV MTP 400 mg/m^2^; PO PDN 2 mg/kg	At 3 months: 9/11 (81%) IMP; After 6 months: 11/11 (100%) had seizure recurrence and 7/11 (63%) needed HS	At 3 months: 5/11 (45%) partial IMP; 2/11 (18%) complete IMP; After 6 months: 2/11 (18%) partial IMP	NA
Takahashi et al. (2013) [57]	21	8.2	MTP 30 mg/kg/d (children) to 1 g/d (adults) for 3 days	1/21 (5%) seizure-free; 17/21 (81%) seizure IMP	16/21 (76%) cognitive stabilization; 2/21 (9%) motor IMP; 2/21 (9%) motor worsening	13/21 (62%) discontinued regular pulse therapy
Pellegrin et al. (2021) [79]	40	6.5	PO PDL 2 mg/kg/d to a maximum of 60 mg/d	33/40 (70%) seizure IMP	NA	The study total number of participants was 53, but only 40 were in CTC therapy.
Intravenous Immunoglobulins						
Walsh et al. (1991) [80]	2	9	0.2 mg/kg/d	IMP	Neurological IMP	NA
Klivity et al. (1994) [81]	1	NA	0.2 mg/kg/d	IMP	Neurological IMP	NA
Krauss et al. (1996) [77]	1	15	0.4 mg/kg/d	No effect	No effect	NA
Wise et al. (1996) [81]	1	14	0.4 mg/kg/d	IMP	Motor and cognitive stabilization	NA
Leach et al. (1999) [82]	2	36	0.4 mg/kg/d	IMP	Motor and cognitive stabilization	NA
Vinjamuri et al. (2000) [83]	1	39	0.4 mg/kg/d	IMP	Motor and cognitive stabilization	NA
Villani et al. (2001) [84]	1	18	0.4 mg/kg/d	IMP	Slight Motor and cognitive IMP	NA
Granata et al. (2003) [12]	11	5.8	2 g/kg	3/11 (27%) seizure IMP	1/11 (9%) major IMP; 2/11 (18%) minor IMP	NA
Arias et al. (2006) [85]	1	51	0.4 mg/kg/d	IMP	Neurological (motor and language) IMP	
Kupila et al. (2011) [86]	1	41	0.4 mg/kg/d	EPC ceased	Neurological IMP	NA
Bien et al. (2013) [87]	7	5.3	0.4 mg/kg/d	1/7 (14%) worsening	Motor and cognitive stabilization	NA
Takahashi et al. (2013) [57]	13	13.6	0.4 mg/kg/d	0/13 (0%) seizure-free; 3/13 (23%) seizure IMP	6/13 (46%) cognitive stabilization; 2/13 (15%) motor IMP; 8/13 (62%) motor worsening	NA
Hoffman et al. (2016) [88]	8	NA	NA	3/8 (37%) transient IMP	NA	NA
Castellano et al. (2017) [89]	3	47	NA	1/3 (33%) IMP	1/3 (33%) cognitive IMP	NA
Pellegrin et al. (2021) [79]	35	NA	2 g/kg over 2 to 5 days monthly	14/35 (40%) seizure IMP	NA	NA
Jaafar et al. (2023) [90]	7	9	2 g/kg over 2 to 5 days monthly	3/7 (43%) seizure-free 2/7 (29%) IMP	1/7 (14%) cognitive IMP; 4/7 (57%) stable motor function; 1/7 (14%) IMP in motor function	NA
Plasmapheresis/Immunoadsorption						
Rogers et al. (1994) [91]	1	9	NA	IMP with short-term recurrence	IMP with short-term recurrence	Anti-GluR3(+)
Andrews et al. (1996) [92]	4	7	NA	3/4 (75%) IMP with rapid recurrence between sessions	2/4 (50%) IMP with rapid recurrence between sessions	3/4 (75%) anti-GluR3(+). AE: 1/4 (25%) infection and thrombosis.
Palcoux et al. (1997) [93]	1	4	NA	Transitory IMP	Transitory IMP	Anti-GluR3(+). AE: infection.
Antozzi et al. (1998) [94]	1	11	NA	IMP	Cognitive and motor IMP	Anti-GluR3(+)
Granata et al. (2003) [12]	8	6.3	NA	1/5 (20%) EPC ceased with PLEX; 1/3 (33%) seizure IMP with adsorption	0/5 (0%) PLEX; 1/3 (33%) adsorption	5 PLEX + 3 adsorption. 2/8 (25%) anti-GluR3(+). All patients with improvement were anti-GluR3(+)
Thilo et al. (2009) [95]	1	13	NA	Seizure IMP with short-term recurrence	NA	Anti-GluR3(−)
Amrom et al. (2014) [96]	1	8	NA	No effect	No effect	Lupus-associated
Sanfilippo et al. (2016) [97]	1	30	NA	Seizure-free after 1 session	Stabilization of brain atrophy and IMP of hypersignal after 1 session	Anti-GluR3(−)
Timarova et al. (2016) [98]	1	17	NA	No effect	NA	Anti-ganglioside GQ1b(+) and GAD(+)
Castellano et al. (2017) [89]	1	53	NA	No effect	No effect	Adult-onset
Soh et al. (2017) [99]	1	36	NA	No effect	No effect	Adult-onset
Stabile et al. (2018) [100]	2	19.5	NA	No effect	No effect	Adult-onset
Sansevere et al. (2020) [101]	1	6	NA	No effect (HS)	No effect	Psoriasis and uveitis
Cantarín-Extremera et al. (2020) [102]	1	17	NA	Transitory effect (recurrence at 1 month)	NA	Anti-GluR3(−)
Pellegrin et al. (2021) [79]	2	NA	NA	NA	NA	NA
Tacrolimus						
Bien et al. (2004) [4]	7	11.6	0.2–0.8 mg/kg (serum level 5–15 ng/L)	No effect	5/7 (71%) stabilization and 1/7 (14%) motor IMP. Slowing of brain MRI atrophy	NA
Thilo et al. (2009) [95]	1	13	Up to 16 mg/day	No effect	NA	NA
Terra-Bustamante et al. (2009) [103]	1	NA	NA	No effect	NA	NA
Takahashi et al. (2013) [57]	12	8.8	Starting dose 0.1 mg/kg/d (children) or 3 mg/d (adults) with dose escalation based on blood levels	1/12 (8%) seizure-free; 5/12 (42%) responders	9/12 (75%) cognitive stabilization; 1/12 (8%) motor IMP	NA
Bien et al. (2013) [10]	9	5.7	12–15 ng/L, serum level achieved	No effect	Motor worsening and brain MRI atrophy less marked than historical cohort	AE: 2 severe infections
Liba et al. (2017) [104]	1	7	NA	No effect	No effect	NA
Vyas et al. (2021) [14]	2	9.5	0.2 mg/kg/day	2/2 (100%) IMP	2/2 (100%) motor IMP	NA
Fukuoka et al. (2022) [105]	1	8	NA	IMP	No effect	NA
Trapp et al. (2024) [106]	1	4	NA	IMP and EPC stop	Motor and cognitive stabilization	NA
Mycophenolate Mofetil						
Thilo et al. (2009) [95]	1	13	2 g/d	No effect	No effect	Anti-GluR3(−)
Liba et al. (2015) [107]	1	8	MPM + CTC + cyclophosphamide	Seizure-free	No neurological decline	ANA 1/640
Liba et al. (2017) [104]	1	7	NA	No effect	No effect	RE classical form
Garg et al. (2019) [108]	1	29	MPM 3 g per day + CTC	No seizure	No neurological decline	NA
Orsini et al. (2019) [109]	1	6.5	750 mg/m^2^	Seizure IMP	Neurological IMP	NA
Azathioprine						
Muto et al. (2010) [110]	1	13	NA	No effect	No effect	Uveitis
Bittner et al. (2013) [87]	1	8	NA	No effect	No effect	Typical form of RE
Lagarde et al. (2016) [111]	1	37	NA	No effect	No effect	Uveitis
Klaa et al. (2020) [112]	1	11	100 mg	Partial efficacy	Mild IMP in motor function	Typical form of RE
Pellegrin et al. (2021) [79]	30	6.8	1.5 mg/kg/day PO; only children	25/30 (83%) seizure IMP	No clear effect	A total of 53 patients, of which 30 were in use of azathioprine. Individuals enrolled after CTC therapy. AE: 3 leukopenia (1 stop).
Rituximab						
Laxer et al. (2008) [113]	9	NA	NA	3/9 (33%) seizure-free; 5/9 (55%) seizure IMP	8/9 (89%) motor or cognitive IMP	NA
Thilo et al. (2009) [95]	1	13	NA	Seizure-free for six months. After, seizure Recurrence, but it was less severe	No effect	Anti-GluR3(−)
Bittner et al. (2013) [87]	1	8	NA	No effect	No effect	NA
El Tawil et al. (2016) [114]	1	43	NA	Seizure IMP	Motor and language IMP	NA
Timarova et al. (2016) [98]	1	17	NA	Seizure IMP	NA	Anti-ganglioside GQ1b(+) and GAD(+)
Schneider-Hohendorf et al. (2016) [115]	1	12	NA	No effect	No effect	NA
Liba et al. (2017) [104]	1	7	NA	No effect	No effect	NA
Castellano et al. (2017) [89]	2	NA	NA	No effect	No effect	Adult onset
Sansevere et al. (2020) [101]	1	6	NA	No effect (HS)	No effect	Psoriasis and uveitis
Cantarín-Extremera et al. (2020) [102]	1	17	NA	No effect	No effect	Anti-GluR3(−)
Adalimumab						
Lagarde et al. (2016) [111]	11	6.5	24 mg/m^2^ with a maximum of 40 mg via SC every 14 days	5/11 (45%) responders (no seizure free)	3/5 (60%) motor IMP. 3/5 (60%) with cognitive stabilization.	AE: 1 superficial skin infection.
Anakinra						
Mochol et al. (2021) [116]	1	17	SC 100 mg	Seizure-free	NA	AE: pneumonia/ urinary infections.
Arcan et al. (2024) [117]	1	38	SC 100 mg	Seizure IMP	Motor IMP	AE: no side effects.
Cyclophosphamide						
Krauss et al. (1996) [77]	1	15	750 mg/m^2^	Transitory effect for 6 months then worsening	Transitory effect for 8–9 months then worsening	NA
Granata et al. (2003) [12]	4	5.5	500 mg/m^2^	No effect	No effect	AE: 1/4 (25%) severe leukopenia.
Amrom et al. (2014) [96]	1	8	NA	No effect	No effect	Lupus-associated
Liba et al. (2015) [107]	1	6	CPP and CTC and MPM	Seizure-free (15 months)	NA	ANA 1/640
Timarova et al. (2016) [98]	1	17	NA	No effect	NA	Anti-ganglioside GQ1b(+) and GAD(+)
Soh et al. (2017) [99]	1	36	NA	No effect	No effect	Adult-onset
Stabile et al. (2018) [100]	2	19.5	NA	No effect	No effect	Adult-onset
Mitoxantrone						
Stabile et al. (2018) [100]	2	19.5	NA	EPC ceased and seizure IMP	Stabilization	Adult-onset. AE: leukopenia.
Natalizumab						
Bittner et al. (2013) [87]	1	8	Monthly cycles of natalizumab (300 mg)	EPC ceased and seizure IMP	NA	NA
Soh et al. (2017) [99]	1	36	NA	No effect	No effect	Adult-onset
Ganciclovir						
McLachlan et al. (1993) [118]	1	24	IV ganciclovir	Seizure IMP	Stabilization	NA
McLachlan et al. (1996) [119]	4	9.9	IV ganciclovir 10 mg/kg	2/4 (50%) seizure IMP; 1/4 (25%) no effect	1/4 (25%) neurological IMP	NA
Vadlamudi et al. (2000) [120]	1	54	IV ganciclovir	Transient IMP	Transient IMP	NA
Other Therapies						
DeToledo et al. (1994) [121]	1	14	PO zidovudine 800 mg/d	Seizure IMP	Stabilization	Side effects prevent reattempt of zidovudine therapy. AE: granulocytopenia.
Marjanovic et al. (2003) [122]	1	4.5	Thalidomide 300 mg/d	Seizure IMP	Stabilization	Anti-GluR3(+)

Abbreviations: AAO, age of acute stage onset; AE: adverse effect/ adverse event; ANA, antinuclear antibody; CPP, cyclophosphamide; CTC, corticosteroid; EPC, epilepsia partialis continua; GAD, anti-glutamic acid decarboxylase; HS, hemispherectomy/ hemispherotomy; IMP, improvement/ improved; IV, intravenously; MPM, mycophenolate mofetil; MTP, methylprednisolone; NA, not available/ not reported/ not detailed; PDN, prednisone; PDL, prednisolone; PLEX, plasmapheresis; PO, per oral/ per mouth; SC, subcutaneously; (+), present; (−), absent. ^I^ The number of patients included in the study. ^II^ Mean age (in years) of onset of the patients with RE in the study. ^III^ Seizure efficacy with medical therapy, including decreased frequency of seizure episodes. ^IV^ Neurological manifestations efficacy with medical therapy other than epilepsy and EPC.

**Table 6:** Seizure outcomes in children with Rasmussen’s encephalitis undergoing resective or hemispheric epilepsy surgery.

Reference	N ^I^	Age ^II^	Sex ^III^	ASO ^IV^	Op	Seizure Free	Follow-Up
Honavar et al. (1992) [141]	19	13.2	12	7.2	HS, RS	8/19 (42%)	<1–15
Villemure et al. (1993) [142]	9	6.9	6	3.2	HS	7/9 (78%)	1–17 years
Döring et al. (1999) [143]	4	8.8	NA	4.8	HS	4/4 (100%)	<5 years
Topçu et al. (1999) [144]	6	9.2	3	7.1	LB, RS	1/6 (17%)	1–5 years
Sinclair et al. (2004) [145]	3	10.7	2	NA	HS, RS	2/3 (67%)	1–10
Korkman et al. (2005) [146]	4	6.1	3	NA	HS	3/4 (75%)	2 years
Tubbs et al. (2005) [147]	5	9.8	NA	4	HS	5/5 (100%)	13–23
Battaglia et al. (2006) [148]	1	4.5	1	2	HS	1/1 (100%)	6 years
Bahi-Buisson et al. (2007) [78]	11	9.5	6	6	HS	4/11 (36%)	<1–8 years
Delalande et al. (2007) [149]	25	12.3	NA	5.8	HS	16/20 (80%)	0–10 years
Liu et al. (2007) [150]	2	9.5	NA	NA	RS	1/2 (50%)	3–8 years
Chandra et al. (2008) [151]	8	6.1	2	2.3	HS	8/8 (100%)	1–4 years
Ramesha et al. (2009) [125]	10	11.6	4	6.4	HS, RS	7/10 (70%)	1–10 years
Terra-Bustamante et al. (2009) [103]	25	7.7	12	5.4	HS, RS	11/22 (50%)	1–13 years
Caraballo et al. (2011) [152]	13	14.1	5	7	HS	NA	8–14 years
van Schooneveld et al. (2011) [153]	4	11.6	3	8	HS	2/4 (50%)	2 years
Hamad et al. (2013) [154]	9	6.9	NA	NA	HS	8/9 (89%)	1–9 years
Takahashi et al. (2013) [57]	17	NA	NA	NA	HS	6 (35%)	NA
Villarejo-Ortega et al. (2013) [155]	3	7.6	1	5.4	HS	3/3 (100%)	2–5 years
Granata et al. (2014) [156]	16	11.5	8	6.1	HS	8/11 (73%)	3–20 years
Guan et al. (2014) [157]	20	8.9	11	5.7	HS	12/15 (80%)	3–18 years
Wang et al. (2014) [158]	7	9	3	NA	HS	2/2 (100%)	9–21 years
Casciato et al. (2015) [136]	5	28.2	4	23	RS	1/5 (20%)	1–6 years
Hoffman et al. (2016) [88]	13	10.6	7	NA	HS, RS	5/7 (71%)	1–10 years
Guan et al. (2017) [159]	45	8.0	16	5.7	HS	34/45 (75%)	<1–8 years
Bellamkonda et al. (2020) [138]	41	8.8	18	5.9	HS	28/41 (68%)	At 1 year follow-up. 48% and 22% of the patients at 5 and 10 years, respectively.
Sundar et al. (2022) [160]	30	8.6	17	4.7	HS, RS	25/30 (83%)	At 1 year follow-up. 63.6% and 55.6% of the patients at 5 and 10 years, respectively.
Thomé et al. (2024) [161]	44	9	23	6	HS	28/41 (68%)	0.2–23 years

Abbreviations: ASO, age of seizure onset; HS, hemispherectomy; LB, lobectomy; NA, not available/not reported/not detailed; Op, operative procedure; RE, Rasmussen’s encephalitis; RS, resection. ^I^ Number of patients with RE assessed in the study. ^II^ Mean age (in years) of the patients with RE at the time of surgical procedure. ^III^ Number of patients with RE that were females. ^IV^ Mean age (in years) of seizure onset of the patients with RE undergoing to epileptic surgery.

**Table 7:** Cognitive outcome after hemispherectomy in patients with RE.

Reference	TP	N ^I^	RH ^II^	Age ^III^	AAO ^IV^	FU ^V^	Specific Comments Regarding Cognition to the Population with RE
Vargha-Khadem et al. (1991) [163]	6	4	2	12.2	8.4	4.1	None.
Caplan et al. (1996) [164]	4	4	4	13.3	8.4	4.1	Improvement in reasoning, language, and social communication were associated with a short delay between the onset of seizures in RE and the HS.
Vining et al. (1997) [165]	58	27	16	9.7	NA	6.7	Recommended early HS in patients with RE. They found that intellectual efficiency can improve after HS.
Stark et al. (1997) [166]	2	2	1	3.1	1.7	2	L HS does not improve language impairment. RH can improve language function.
Boatman et al. (1999) [167]	6	6	0	10.3	7.4	1	L HS can sustain language ability. Comprehension was related to the short delay between the seizure onset in RE and the HS. Expressive functions do not change with HS.
Curtiss et al. (2001) [168]	43	10	4	8.4	5.1	6.1	Late AAO and late age at the surgery positively correlate with language outcome, but only in the cases of RH.
Telfeian et al. (2002) [169]	1	1	0	16	11	2.5	HS can be associated with favorable language outcomes.
Hertz-Pannier et al. (2002) [170]	1	1	0	9.0	5.5	1.5	Receptive functions, compared to expressive, have better outcomes.
Devlin et al. (2003) [171]	33	4	NA	8.1	4.2	NA	Recommended early surgery in RE, despite complications of cognitive and motor impairments.
Trudeau et al. (2003) [172]	1	1	0	17	5	0.2	L HS does not affect expressive and receptive domains. It is associated with poor reading and writing abilities.
Pulsifer et al. (2004) [173]	71	37	21	9.2	6.0	5.7	Language is more impaired after L HS than RH.
Jonas et al. (2004) [174]	115	21	9	7.8	4.9	2	HS improves development. From 11% (preoperatively) to 29% (postoperatively) achieving good development.
Delalande et al. (2007) [149]	83	25	16	12.6	5.8	4.4	Favorable seizure and behavioral outcomes are observed after HS.
Liégeois et al. (2008) [175]	30	8	4	NA	6.5	5.8	None.
Terra-Bustamante et al. (2009) [103]	25	23	12	7.7	5.4	5.3	Favorable seizure control. Cognitive decline was observed in more than a third of the patients after HS. Patients with an L RE show persistent language deficits.
Thomas et al. (2010) [176]	16	9	NA	NA	NA	NA	No differences in language skills are observed between L HS and RH.
Moosa et al. (2013) [177]	115	10	NA	NA	NA	6.1	Patients with RE, compared to other etiologies, present better language outcomes.
Ramantani et al. (2013) [178]	52	6	NA	7.0	4.2	3.3	Favorable preoperative scores are observed in RE but did not improve after HS. HS should be considered independent of age.
Villarejo-Ortega et al. (2013) [155]	17	3	1	7.6	5.4	3.1	No cognitive deterioration was observed after HS. Favorable seizure and functional outcomes are related to short-delay HS.
Granata et al. (2014) [156]	16	16	12	11.5	6.1	10	Favorable seizure and functional outcomes are related to short delay HS.
Guan et al. (2014) [157]	20	20	14	8.9	5.7	5.5	Favorable seizure and cognitive outcomes are related to short-delay HS. All patients presented normalized language functions after HS.
Bulteau et al. (2015) [179]	6	6	0	6.1	4.8	5.6	Favorable language outcome after HS. L HS is associated with good lexico-semantic recovery. There is no change in syntactic and phonological scores.
de Bode et al. (2015) [180]	10	3	0	6.0	4.7	6.9	RH can potentially support grammatical abilities in the case of prenatal insult.
Gröppel et al. (2015) [181]	28	1	1	3.5	2	3.0	Improvement of language function after HS.
Grosmaitre et al. (2015) [182]	1	1	0	6.9	5	4.1	L HS improved intellectual and language outcomes.
Hoffman et al. (2016) [88]	13	13	8	10.6	NA	5.6	Good seizure control after HS. Long-term benefits were observed for cognitive and language functions.
Save-Pédebos et al. (2016) [183]	40	13	2	8.1	6.9	7.5	None.
van Schooneveld et al. (2016) [184]	31	7	3	NA	NA	NA	None.
Bulteau et al. (2017) [185]	12	8	5	11.7	6.7	5.9	None.
Guan et al. (2017) [159]	45	45	23	8.0	5.7	2.6	Favoravable seizure outcome after HS. Also, the favorable outcomes are related to short-delay HS.
Wilson et al. (2017) [186]	25	10	3	NA	NA	9.0	None.
Rudebeck et al. (2018) [19]	21	21	12	NA	6.3	1.5	Verbal functions are more impaired in L HS than in RH. There is a decrease in verbal and nonverbal functions after HS. A favorable outcome is related to short delay HS.
Kliemann et al. (2019) [187]	6	3	3	7.3	6.3	18.2	None.
Nahum et al. (2020) [188]	205	96	NA	NA	NA	6.1	Deficits are more commonly observed in L HS than RH.
Silva et al. (2020) [189]	15	6	2	9.3	6.8	3.1	None.
Tavares et al. (2020) [190]	13	4	NA	13.8	6.7	1.1	None.
Kliemann et al. (2021) [191]	4	1	1	20	11	12.8	None.
Shurtleff et al. (2021) [192]	71	6	NA	NA	NA	NA	Normal brain biopsy is associated with better cognitive functions.
Sousa et al. (2021) [193]	1	1	0	17	10	1.3	None.
Borne et al. (2022) [194]	3	3	0	9.3	7.8	11.1	Favorable cognitive recovery is noticed with L HS, especially executive functions.
Liu et al. (2022) [195]	40	40	22	8.7	NA	2	A favorable outcome is related to short-delay HS and normal brain biopsy. Cognition is more affected in the L HS than in RH.
Pinabiaux et al. (2022) [196]	40	13	2	8.1	6.9	7.5	None.

Abbreviations: AAO, age of acute stage onset; FU, follow-up; HS, hemispherectomy; L, left; NA, not available/not reported/not detailed; RE, Rasmussen’s encephalitis; TP, total population. ^I^ Number of patients with RE included in the study. ^II^ Number of patients with RE undergoing right hemispherectomy. ^III^ Mean age (in years) of the patients with RE when hemispherectomy was performed. ^IV^ Mean age (in years) of acute stage onset of the patients with RE undergoing hemispherectomy. ^V^ Mean follow-up (in years) for all the population from the study.

**Table 8:** Differential diagnosis of RE and explorations, adapted from Bien et al. (2005) [4].

Differential Diagnosis	Clinical and Laboratory Criteria
Unihemispheric epileptic syndromes	
Cortical dysplasia, hemimegalencephaly, tuberous sclerosis, Sturge-Weber-syndrome, stroke, tumor Hemiconvulsion-hemiplegia-epilepsy-syndrome	Focal seizures in infancy or early childhood MRI with gadolinium No progression on MRI Usually occurring in infancy, initial (tonic-) clonic unilateral seizures presenting as status epilepticus Early MRI: diffuse cytotoxic edema of the whole hemisphere Hemiparesis, hemiatrophic hemisphere on MRI, and focal epilepsy
Epilepsia partialis continua (EPC) due to metabolic disorders	
Diabetes mellitus: ketotic/ non-ketotic hyperglycemia, type I diabetes and anti-GAD-65-antibodies, renal or hepatic encephalopathy	History, blood tests, anti-GAD-65-antibodies
Metabolic or degenerative progressive neurological diseases	
MELAS and other mitochondriopathies; Alpers syndrome or polymerase gamma-related disorders	Blood-lactate, mitochondrial DNA genetic testing for mutations, muscle biopsy, progressive illness, EEG: bilateral abnormalities, MRI; T2 hyperintensity in the occipital lobes and thalami, liver impairments. Autosomal recessive mutations in the DNA Polymerase gamma, catalytic subunit (POLG) gene
Inflammatory/infectious diseases	
Cerebral vasculitis in systemic connective tissue disease (e.g., lupus erythematosus)	History, other clinical features, blood abnormalities (ANA, ANCA), antiphospholipid antibodies (anticardiolipin antibody, anti-beta-2-glycoprotein-I (aβ2GPI) antibody) anti-Ro/SSA antibody anti-double-stranded DNA (anti-dsDNA) antibodies anti-thyroglobulin, anti-thyroid peroxidase
Autoimmune encephalitis; Antibodies: autoimmune encephalitis with intracellular antigens; autoimmune encephalitis with cell-surface antigens; paraneoplastic syndrome	History, other clinical features, movement disorders, psychiatric features Autoantibodies (blood and CSF), anti-neuronal antibodies, anti-GQ1b, GD1b, GM1, GM2, anti-HU (a,b)
Subacute sclerosing panencephalitis and other delayed subacute measles encephalitis with or without immunodeficiency	History, vaccination status, early measles, EEG: periodic discharges, measles-antibodies in CSF
Multiple sclerosis	History of previous episodes, additional deficits, MRI; CSF: oligoclonal bands
Infectious diseases	Antibody tests: HIV, HSV1, HSV 2, Syphilis, CMV, EBV, HHV6, mycoplasma, chlamydiae, Borrelia burgdorferi, Bartonella henselae

Abbreviations: CSF, cerebrospinal fluid; EEG, electroencephalogram; MRI, magnetic resonance imaging.

**Table 9:** Neuroimaging of some cerebral hemi-atrophy causes.

Conditions and Reference	Pathophysiology	Clinical Presentation	Neuroimaging Features		
			Parenchymal	Ventricular	Calvarial
Dyke-Davidoff-Masson syndrome [198]	Brain damage during fetal life or early childhood	Seizures, contralateral hemiparesis, facial asymmetry, cognitive disabilities	Unilateral cerebral atrophy	Dilated ventricle on the same side	Calvarial thickening on the same side
Fishman syndrome [199]	Phakomatosis. Mutations in the FGFR1 gene	Lipomas of the cranium, face, and neck, ipsilateral lipodermoids of the eye, and ipsilateral brain anomalies	Unilateral cerebral atrophy	Dilated ventricle on the same side	Usually, absent
Neurofibromatosis type I [200]	Phakomatosis. Neurofibromin 1 gene	Neurofibromas, optic gliomas, and cafe-au-lait spots	Unilateral cerebral atrophy	Dilated ventricle on the same side	Sphenoid dysplasia
Rasmussen encephalitis	Chronic encephalitis	Drug-resistant epilepsy, history of viral fever	Unilateral cerebral atrophy	Dilated ventricle on the same side	Absent
Sturge-Weber syndrome [201]	Phakomatosis associated with anomalous development of cortical veins	Facial port wine stains, seizures, hemiparesis, development delay	Unilateral cerebral atrophy	Dilated ventricle on the same side	Usually calvarial thickening
Unilateral megalencephaly [202]	Hamartomatous overgrowth of an attire hemisphere related to neuronal proliferation abnormalities	Focal or generalized spasms, development delay, hemiparesis	Unilateral cerebral enlargement	Enlarged ventricle on the same side	Absent

## Data Availability

No new data were created or analyzed in this study.
